# Multistakeholder Perspectives on the Determinants of Family Fundamental Movement Skills Practice: A Qualitative Systematic Review

**DOI:** 10.3390/children11091066

**Published:** 2024-08-30

**Authors:** Robert J. Flynn, Andy Pringle, Clare M. P. Roscoe

**Affiliations:** Clinical Exercise and Rehabilitation Research Centre, School of Sport and Exercise Sciences, University of Derby, Kedleston Road, Derby DE22 1GB, UK; a.pringle@derby.ac.uk (A.P.); c.roscoe@derby.ac.uk (C.M.P.R.)

**Keywords:** fundamental movement skills, physical activity, children, parents, family, determinants, perspectives

## Abstract

Background: Childhood obesity is a significant public health crisis that is exposing children to associated morbidities and premature mortality. However, parents can positively influence physical activity trajectories and improve health outcomes by nurturing fundamental movement skills (FMS) in children. This is the first study to explore the determinants of family FMS practice via a systematic synthesis of qualitative evidence. Methods: Keyword searches were completed in SPORTDiscus, PubMed, Scopus, Web of Science, and Embase. Studies that offered perspectives relating to influences on the FMS of 2–6-year-old children in the family context via qualitative approaches, including visual methodologies that provided an important voice to children, were included. A thematic analysis was used to establish key themes. Results: The emergent themes included parent knowledge and beliefs, self-efficacy of parents to teach, and the home environment. Parents often undervalued FMS and lacked the self-efficacy to teach due to poor understanding, conflicting priorities, and multifaceted societal influences. Children preferred autonomous play and socialisation but were negatively influenced by technology and restrictive household rules. Conclusions: Greater knowledge exchange between stakeholders is necessary to empower parents and enhance FMS application at home. More community initiatives could facilitate greater access to outdoor spaces, facilities, and equipment, which may improve family engagement with FMS.

## 1. Introduction

Children born in the 21st century will be the first generation in modern memory for whom life expectancy falls and, on average, will live shorter lives than their parents [1,2]. This prediction is intertwined with an ever-growing and unrelenting global childhood obesity epidemic [3]. One in three children in the United Kingdom (UK) leave primary school overweight or obese [4], and there are now more than 100 million obese children worldwide [5]. Childhood obesity has doubled over the last four decades and continues to proliferate in both frequency and severity [6], exposing children to the long-term risk of severe disease states and premature mortality [5]. This situation has prompted the World Health Organization (WHO) to declare childhood obesity as one of the most serious public health challenges of the 21st century [7]. At the heart of this rise in obesity lies issues surrounding motor competence and physical activity (PA) participation [8]. Research has indicated a shift in children’s PA patterns over time, now in favour of extended periods of sedentarism [9,10]. Further, sedentary behaviour has been shown to be both a potential cause and consequence of obesity, and poor competence in fundamental movement skills (FMS) is thought to be a crucial factor in this relationship [11].

FMS are rudimentary abilities in children, commonly divided into three constructs of locomotion, object control, and stability [12]. Locomotor skills include activities such as running and leaping that propel the body across a space [13]; object control skills allow the coordinated manipulation of an object, such as kicking or striking a ball [14], and stability skills involve exercises of balance and postural control such as standing on one foot [15]. These elementary faculties act as building blocks for the development of more complex movements to support activities of daily living and improve a child’s capacity to meaningfully engage in play, games, and sports [16,17,18]. Moreover, FMS are known to be a key element of children’s overarching physical literacy, providing the confidence, motivation, and aptitude for purposeful and lifelong PA participation [19]. This positive interaction is considered to be a crucial aspect of obesity prevention [20] and is associated with a plethora of physical, psychosocial, and general health benefits in childhood and later life [21]. Children acquire basic movement patterns rapidly throughout a critical window of biological maturation during the early years (EY) of childhood (2–5 years) [17]. Therefore, it is vital that children are provided with developmentally appropriate environments and practice opportunities during early childhood to fully assimilate FMS [22] and lay the foundations for healthy PA participation.

Despite the well-known health-enhancing qualities of FMS, recent data have indicated a concerning secular decline in children’s motor competence, both domestically [23] and internationally, [24] resulting in a failure to provide a suitable foundation for children to adequately participate in PA [25]. In the UK, FMS have failed to meet expected levels in both EY children [16,26,27] and children in later age groups [28,29,30]. Disturbingly, comparable and consistently below average findings have also been conveyed in European [31,32,33] and international youth [34,35,36]. In a reciprocal manner, it has been estimated that less than 10% of EY children and under half of young people in the UK meet the established governmental guidelines for PA [37] and, globally, children’s PA engagement has been graded as poor [38,39]. This negative spiral appears to manifest in early childhood [9] and track into older age groups [25] in spite of the myriad of FMS interventions that have targeted EY educational settings previously [19,40]. Given the ongoing inadequacies surrounding children’s FMS and PA, a change of approach may be warranted in order to more effectively intervene in EY children’s FMS.

Parents and caregivers have been described previously as *gate keepers* since they serve as primary role models to their children and possess the power to positively influence their children’s PA behaviours [41]. Equally, parents can provide children with nurturing and supportive home environments full of positive movement opportunities that are conducive with learning FMS [42]. Children spend much of their free time in the company of family, so parents present a unique opportunity to improve children’s motor competence [43]. This concept has been supported by a recent systematic review that communicated significant gains in children’s FMS through parental engagement with motor skill interventions [44]. Notably, interventions that have initiated parent–child co-activity [1,45], or have empowered parents through educational workshops [46,47], or have provided digital input via mobile applications [48,49], have all proven to be effective in ameliorating children’s FMS. In contrast, similar family-focused interventions have failed to recreate the same success [50,51,52], possibly due to their use of indirect methodologies and a lack of structured FMS guidance to support family engagement [53]. However, FMS remain underexplored in the context of family [54], and there is currently insufficient understanding of the social and environmental factors that conclusively determine what may foster or inhibit motor competence in the home environment [55]. Consequently, further research is needed to correctly identify the determinants of family FMS participation to ensure more effective promotion and strategy development.

To date, the literature has been dominated by quantitative approaches that have explored the socioecological correlates of FMS (interrelations between personal and environmental factors that influence human behaviour within a broader context) [21,26,56,57,58,59,60]. Recently, it has been identified that parent caregiving may be superior to grandparent caregiving in terms of FMS development [61]. Furthermore, children of low socioeconomic status are more likely to have lower FMS aptitude than their more affluent peers [56,62]. Parents’ educational status [60], parental endorsement of PA and active transport [21,62,63], ethnicity, native language, cultural background [26,56], and screen time [64] have all been associated with children’s FMS outcomes in the context of family. However, the established correlations often prove inconsistent and are not always upheld by comparable research [57,59], nor do these patterns provide a complete appreciation of the multitude of variables that exist within complex family dynamics that may dictate the motor development of children [65]. Conversely, qualitative research methods can play an important role in the collection of in-depth perceptions from relevant stakeholders [66] and may be a valuable method of exploring unmapped influences on family engagement with FMS.

A small number of qualitative studies have thus far gathered important perspectives from parents [67] and educators [68] on the determinants of family FMS practice and identified potential issues surrounding parental knowledge and understanding of FMS. Additionally, EY children who are central to the phenomenon, have themselves provided further insight into what makes PA enjoyable via the developmentally appropriate draw and tell technique [69]. However, the existing qualitative evidence is extremely limited. In comparison, qualitative approaches have been extensively utilised to examine children’s PA and sedentary behaviours [9,70,71,72]. But these studies have approached PA in the broadest sense and have failed to consider the role of FMS or the factors that may influence FMS engagement. The current research gap is concerning given the critical role FMS play in children’s PA promotion [25], especially considering recent research which has suggested that significantly fewer parents intend to support FMS at home compared to PA [73]. This further highlights the urgent need to gain greater collective perspectives and understanding of the specific needs of families in terms of FMS support. To the best of our knowledge, at the time of writing, no study has collated the determinants of FMS in the family context via a qualitative approach. Therefore, the aim of this study is to systematically synthesise qualitative literature to investigate the key determinants of family FMS practice from a holistic multistakeholder perspective.

## 2. Materials and Methods

### 2.1. Protocol and Registration

The protocol for this systematic review was registered with and approved by the International Prospective Register of Systematic Reviews (PROSPERO) in April 2024. The review protocol can be accessed on the PROSPERO website by searching the registration number CRD42024534565 or via the following address: https://www.crd.york.ac.uk/prospero/display_record.php?ID=CRD42024534565 (accessed on 11 April 2024).

### 2.2. Study Selection Criteria

An exhaustive systematic search of the existing literature was conducted by the lead researcher (RF), in accordance with the standards recommended by the preferred reporting items for systematic reviews and meta-analyses (PRISMA) framework [74], to assemble all peer-reviewed English language articles published worldwide with no date restrictions on publication. The broad criterion regarding the date of publication was important in order to gather as many views and opinions related to FMS in the family context as possible due to a paucity of research in this area. All qualitative studies, or mixed-methods and multi-method approaches that contained a qualitative element, were eligible for inclusion. Studies that reported in-depth discussions on the determinants of family FMS practice at home or in the wider community, or that offered insights into caregiver influences on children’s motor development within the childcare or educational settings, were included. The perceptions ascertained must have been in relation to families of normally developing children aged 2–6 years, as this age range allowed for variations in the preschool period around the world [44]. Input from relevant stakeholders may have been collected through focus group discussions or interviews, or via adapted and developmentally appropriate qualitative methodologies that facilitated meaningful communication with EY children on their active play preferences. For the purposes of this research, stakeholders were considered to be educators, coaches, or childcare providers; primary caregivers consisting of parents, grandparents, or legal guardians, and EY children, since this population were most likely to have had direct experience in this area.

Review articles were not considered. Quantitative studies have been extensively investigated previously, thus were not eligible for this review. Studies were also excluded if perceptions were not related to EY children; if the children were clinically diagnosed with disabilities, morbidities, or co-ordination difficulties; if the views were generalised to PA and not directly reporting on FMS, if the views offered were not related to family FMS practice or caregiver influence on the children’s FMS opportunities away from the home environment; if the qualitative data were derived from or associated with interventions or programme evaluations, which had the potential to introduce bias, or if they were reasoned to be too specific to the particular intervention in question and thus not representative of normal family context.

### 2.3. Search Strategy

An extensive scoping phase was carried out prior to the full search to ensure specificity of the search terms. Subsequently, a comprehensive and tailored literature search of five online databases including Scopus, Web of Science, Embase, PubMed, and SPORTDiscus was completed in May 2024, applying keywords that appeared within the title and abstract with the following Boolean phrases: (“fundamental movement skills” OR “FMS” OR “fundamental motor skills” OR “motor skills” OR “motor competence” OR “motor proficiency” OR “motor ability” OR “active play”) AND (“barriers” OR “facilitators” OR “perceptions” OR “perspectives” OR “preferences”) AND (“children” OR “child” OR “childhood” OR “preschool” OR “preschoolers” OR “early years” OR “kindergarten”). EY children are developmentally unable to comprehend and report on abstract concepts such as movements, or dissociate between PA and play [75], so the term “active play” was included as it was felt that this would increase the sensitivity of the search to also capture children’s voices on the subject. All records that were generated were exported and screened using Rayyan software, which has been accepted as a viable and effective screening tool [76,77] and has been utilised by similar reviews [78].

**Figure 1:**
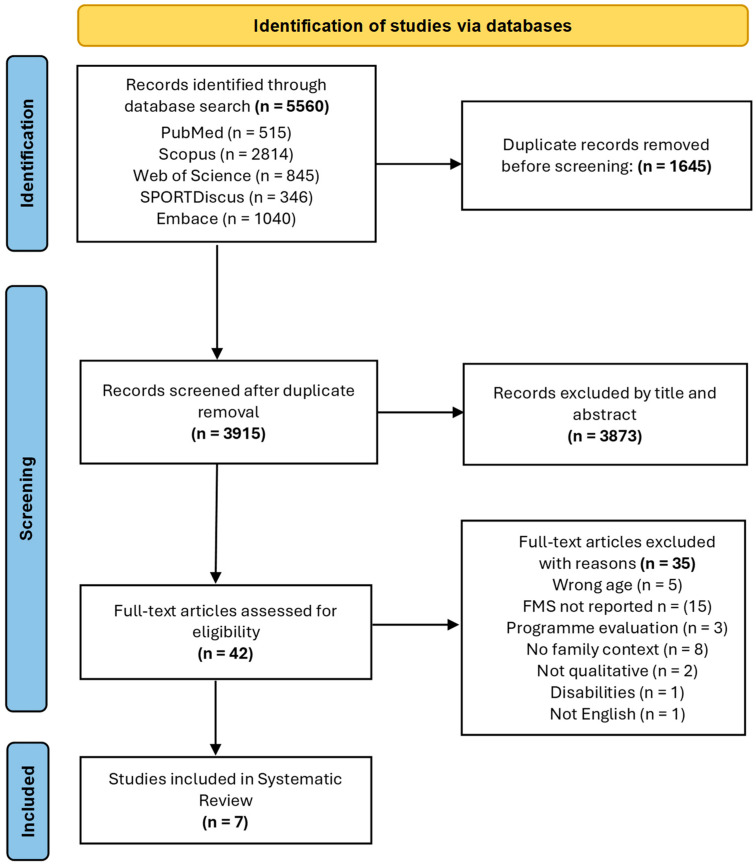
Preferred reporting items for systematic reviews and meta-analyses (PRISMA) flowchart.

Duplicate papers identified by multiple databases were removed. Articles were screened by title followed by an additional screening of the abstracts. In the event of uncertainty as to whether the article had fulfilled the inclusion criteria after screening the abstract, they were included in the full-text screen. Full-text articles were then reviewed for eligibility and all relevant papers were confirmed and put forward for analysis in the review. In addition to this process, reference lists and citations from the full-text articles were manually searched to identify any potentially relevant articles that may have been initially overlooked. However, no additional studies met the review criteria. The full search strategy may be viewed in Figure 1. Five percent of the original search sample was randomly allocated to and examined by the other members of the research team (CR and AP) to ensure there was consensus with the lead researcher’s (RF) decisions. Any studies not initially agreed on were discussed, and a mutual decision was made on inclusion. A total of 95% of the papers were agreed upon in terms of inter-rater reliability. A final search of the literature was conducted in July 2024 prior to final write-up to check for potential updates since the initial search.

### 2.4. Study Quality Assessment

The lead researcher (RF) independently applied the mixed-methods appraisal tool [79] to the studies included in the review to appraise the quality of evidence and identify any potential bias. The studies were first screened using two questions to confirm that they were empirical research, before being assessed according to the criteria that corresponded with the design of their study. Each criterion received a response of “yes”, “no”, or “cannot tell”. A response of “yes” obtained one mark or responses of “no” or “cannot tell” received zero marks. Each study was then awarded an overall quality score ranging from 1* to 5*. For example, if 20% of the quality criteria were met the paper would be attributed with 1* and described as low-quality evidence, whereas if 100% of the criteria were achieved the study would be considered a high-quality 5* paper. The outcomes of the appraisal were shared with the remaining members of the research team (CR and AP) and discussed for added robustness. No disagreements occurred.

### 2.5. Data Extraction and Analysis

The following data were extracted from all eligible articles that fulfilled the inclusion criteria: author(s) and year of publication, country of origin, study design, population sample and characteristics, method of qualitative data collection, mode of analysis, and key themes. The data were analysed via the six-step iterative process of thematic analysis in line with the guidelines first proposed by Braun and Clarke (2006) [80]. The thematic framework approach is a method of identifying, analysing, and reporting patterns and themes that emerge from the transcripts [80], and has been extensively utilised in qualitative research relating to children’s FMS and PA behaviour [81,82,83,84]. All data relevant to the research aim of this review were first extracted and transcribed into a usable form, which was subsequently read and re-read to saturation by RF to ensure familiarisation with the data. Sections of the entire dataset were then highlighted and assigned codes to describe their content, followed by categorisation of the descriptive codes through the identification of patterns and similarities to generate initial themes. These themes were reviewed multiple times to ensure they accurately captured the underlying meaning of the data. Following writing and re-writing multiple times, themes were defined and named to produce a final list of themes, subthemes, and associated quotes. Regular meetings and discussions were held by the research team throughout the process to confirm the themes.

## 3. Results

### 3.1. Study Selection

Overall, 5560 articles were identified using keyword searches across five online search engines. From this total, 1645 articles were confirmed to be duplicates and were removed prior to screening. Subsequently, 3690 articles were excluded based on their title and then a further 183 articles were removed after examination of the abstracts. The most common reasons for exclusion by title and abstract were the studies being on the wrong subject or focused on children diagnosed with disabilities or co-ordinative issues. The 42 articles that remained were read in full and seven articles were included in the final analysis. Reasons for omission after full-text screening included a lack of reporting on FMS, perspectives relating to FMS not being in the context of family practice, or the children involved not being of the correct age group (Figure 1).

### 3.2. Study Quality Assessment

Six of the seven articles were appraised using the qualitative study assessment criteria, and one article under the mixed-methods study criteria. According to the MMAT assessment, six of the articles met 100% of the quality criteria related to their study design and were deemed to be high-quality 5* evidence. The remaining article fulfilled 60% of the criteria and was awarded a 3* quality rating (Table 1) (please see Appendix A, Table A1 for full presentation of MMAT appraisal outcomes).

### 3.3. Origin and Participants

The seven articles included for analysis were produced in five different countries. Three articles were based in the UK, while single studies originated from Australia, Canada, Italy, and the United States of America (USA), respectively. From these seven articles the overall sample available for this review was 166 participants. Five of the seven articles gathered perspectives on the determinants of family FMS practice from an adult population consisting of 88 participants. The mean adult participant number was 18, ranging from 8 to 31 participants per study. Of this adult sample, 61 participants were parents or primary caregivers of EY children, 24 were educators, and three were external physical education providers. The age of the adult population was only stated in one article, in which the average age was 41 years. In contrast, gender was reported in all five articles that gathered adult viewpoints, comprising 80% female and 20% male representation. The ethnic background was communicated in two articles and was predominantly Caucasian. The socioeconomic status of the samples was diverse amongst the articles. Two of the seven articles considered the preferences and determinants of active play from a total of 78 EY children. Of this child population, one study included 49 children and one study included 29 children, 49% of which were girls and 51% were boys, with an average age of 4.5 years. Neither the ethnicity nor socioeconomic background of the children were reported by these articles.

### 3.4. Study Design, Data Collection, and Analysis

Of the seven articles included, six articles were qualitative studies, and one article had a mixed-methods application. From the five articles that focused on adult perspectives, three articles collected qualitative data via semi-structured interviews, and two articles collated views through focus group discussions. The data obtained from adult participants were analysed using thematic analysis in four of the five articles, and with directed content analysis in one article. Both articles that explored EY children’s views on active play employed the same draw and tell visual methodology, which involved children drawing their preferred activities. Subsequently, the children were asked to expand upon these representations during semi-structured interviews to elucidate the barriers and facilitators of their chosen activity. One of these studies analysed children’s data using thematic analysis, whereas the remaining study did not state a formal method of analysis and appeared to be a subjective summarisation of the children’s responses by the authors.

### 3.5. Determinants of Family FMS Practice

The data compiled from the perspectives of key stakeholders relating to the barriers and facilitators of family engagement with FMS produced three main themes: parent beliefs and understanding of FMS, parenting practices, and the home environment. The results are presented below by theme and associated subthemes and are accompanied by interview extracts. A summary is provided in Table 2.

### 3.6. Parent Beliefs and Understanding of FMS

#### 3.6.1. Subtheme: Value Assigned to FMS

There were varying levels of importance assigned to FMS by parents. A selection of parents offered their perspectives on why they held motor skills in high regard by describing some of the potential benefits related to competence. It was suggested that hand–eye coordination, balance, and running were specifically important abilities to help their children navigate through life [67], and that FMS development would help children develop their confidence and willingness to try new and challenging things [73]. One parent described the possible cognitive benefits associated with FMS, which were considered a learning opportunity for their child that *“helps her brain”* [73]. Several parents spoke of the importance of developing good motor proficiency for the establishment of lifelong PA and sports participation and to safeguard their children’s health and wellbeing. Parents explained the following:

*“When I think of motor activities, I always think sports. I do want my children, once they get older, to be involved in a lot of sports just to stay active.”*

[67]

*“I want him to have those opportunities [speaking about physical activities] and not just sit down and watch people and be like ‘well, I can’t do that’ or ‘I didn’t know how to do that’.”*

[73]

In contrast, certain parents displayed a more passive attitude towards FMS and did not view it as being an important aspect of a child’s overall development:

*“It’s not really important if they do or if they don’t know how to kick a ball.”*

[67]

Some parents did not feel it was important to get involved with their children’s FMS unless they were prompted to do so because their child was struggling with a skill, or if they felt their child was at risk of negative health consequences. For example:

*“I think I am not necessarily teaching her unless she takes the initiative to try and do something, or I see she is struggling.”*

[67]

*“Not really [important] unless my child was obese, and I needed to do something about it.”*

[86]

One educator articulated that they had lost much of their faith in parents’ abilities to appropriately support FMS at home, and because of this they had formed the opinion that schools and childcare should assume full responsibility for teaching FMS to children. This perspective had seemingly been influenced by their recent negative experiences with unenthusiastic parents who were disengaged with FMS at home. The educator explained:

*“Sometimes your kids that do enjoy it [PE] don’t get the opportunities when they go home…I think it [FMS] needs teaching in schools to be quite honest, as I said, you don’t always have the enthusiastic parents that want to teach them.”*

[68]

#### 3.6.2. Subtheme: Conflicting Priorities

One explanation for why parents may view FMS as a low priority was offered by one of the educators, who illustrated how some families of higher affluence often committed exclusively to academic rigour over the provision of PA and FMS:

*“She turns round to me and says, ‘my mum says I don’t need friends and I don’t need sport to get into Oxford’.”*

[68]

Educators also reported that work commitments and family dynamics, such as mothers having the responsibility of caring for multiple children at once, could reduce parents’ time and availability to support FMS, which therefore influences its value:

*“[parents] they’re busy, the dad is always at work, never there and the mum they tend to have quite a lot of children so they’re at home on their own with these children.”*

[85]

*“In our household it’s hard to pay attention to one kid at a time to do something with because there are six of them in the house, so it’s very rare that one of us will get one-on- one time with the other children.”*

[67]

An alternative justification for the low value assigned to FMS was the prioritisation of fun over intentional FMS support. Some parents believed that for their children it was more important to *“let them play”* [73] with freedom instead of implementing a formal structure for teaching and assessment:

*“We need balance between testing and letting children move”.*

[86]

*“I mean I’m just having fun. I didn’t think about teaching her motor skills saying like tuck and roll or kick a ball. It’s just like a game. They’re learning from it, but I don’t really think about it like that.”*

[67]

These parent perspectives were seemingly validated by children’s drawings, as almost all the favoured activities that were depicted were unstructured and involved open-ended free play that encompassed fun and flexibility, with no specific learning objective. Examples included *“playing in the sandpit”*, *“playing sticks”*, and *“climbing”* [69]. When the children were asked for their reasoning behind their activity choices, a consistent response was a desire for autonomy:

*“[Why do you like this activity] Because we get to choose […] You can pick whatever you want.”*

[69]

#### 3.6.3. Subtheme: Knowledge and Promotion of FMS

One parent was able to demonstrate an excellent understanding of the subject by providing some accurate definitions and explanations of FMS:

*“Fine motor skills are manipulation, for example, control of a pencil, putting Lego together. Gross or large movements involve coordination of arms and legs and being able to kick a ball.”*

[86]

Overall, parents’ knowledge and understanding of FMS was limited. This was illustrated when multiple parents indicated that motor skills were an *“important part of life”*, but were subsequently unable to provide any specific reasons as to why they were important [67]. Furthermore, many of the parents who appeared to be unsupportive of FMS seemed to have formed this opinion based on incorrect assumptions that stemmed from poor knowledge and awareness. For example, it was not seen as essential to practice FMS because it was believed that skills are acquired naturally when required, with parents stating that *“kids will just figure it out”* [73]. One parent did not feel they needed any specific *“knowledge or skills”* themselves to enable them to support their child’s FMS, rather they just needed to encourage their child [73]. Another parent incorrectly believed *“you might not necessarily use it [FMS] as an adult”* [67]. Notably, numerous parents did not consider it to be appropriate to teach FMS to EY children because the technique would not be attainable at such a young age, so instruction would be more beneficial in later age groups:

*“Children at this age do not acquire any sort of technique for FMS…I think there’s no technique involved in this case, because we are talking about a kid who is three years old, almost four.”*

[73]

*“I feel like, at some point, they’re going to learn how to hop and they’re going to learn how to jump…I feel like the instruction part comes a bit later than three or four.”*

[73]

One parent admitted that despite them having two children *“my background knowledge is not that good on this area”* [86], and felt that the preschool that their child attended should take more responsibility for improving parents’ knowledge of FMS:

*“I feel like nursery staff need to help me promote this.”*

[86]

This opinion seemed to align with one of the educators, who conceded that although parents were not currently providing enough FMS support to children outside of the school setting, this was perhaps because of a lack of education and guidance provided to parents:

*“We need more support from out of school. I don’t think it is put across to parents how important physical development is.”*

[68]

A headteacher agreed that there were *“lots of parents who really want to do the best thing for their child”, so it was “about educating and promoting, but not making parents feel bad about it”* [68]. However, these viewpoints differed from others, with some educators asserting that their time was devoted to promoting this to the preschool children within the setting, and some parents arguing that it was “the responsibility of the parents to improve their knowledge, parents should accept responsibility for their own child’s health and stop blaming others” [86].

### 3.7. Parenting Practices

#### 3.7.1. Subtheme: Positive Approaches and Engagement

Educators believed that parents who were physically active themselves in turn had a positive influence on their children’s PA behaviour and FMS. This was illustrated by educators who told of their experiences with a *“stereotypical group”* of children that came from active households with *“really sporty parents”* who were more likely to receive further PA opportunities and therefore be more *“physically able”* [68]. Some parents highlighted their engagement in child-driven co-activity as being a similarly influential approach at home that helped foster children’s enjoyment for PA and motivation for learning:

*“Just whatever he feels like doing he’ll ask us ‘will you do this with me?’ and we always tell him okay.”*

[67]

*“I will see openings throughout our physical activity where it’s like, oh, she like found a ball and it’s like, okay, well like for the next couple of minutes it was practice during and back and forth.”*

[73]

Parents who felt confident in their ability to teach FMS to their children were able to communicate specific examples and discussed the ways in which they approached teaching certain skills. One parent described a scenario where they were teaching their child how to throw a frisbee by breaking the skill down into manageable sections while demonstrating and describing the skill:

*“And we were like, showing her how to do it. ‘Can you stand like this; you go like this’.”*

[73]

Parents also frequently used reasoned communication to promote FMS to their children and encourage them to persevere with practice. For example, parents would ask their children to *“do your best”* and reminded them that *“the more you practice, the better you’re going to get”*, while offering reassurance that *“when you get out there, all your friends that are your age, they’re learning too”* [67]. Moreover, parents would regularly offer warmth and praise for both attempting and succeeding at a skill:

*“Any sort of success I think should be celebrated, especially when a child is uncomfortable with their competence and their skills.”*

[67]

#### 3.7.2. Awareness of Child Temperament

When interacting with their children, parents displayed awareness of the moderating role of child temperament in FMS practice and discussed the importance of *“knowing their child’s interests”* and *“making activities fun”* [73] to maximise their engagement. According to parents, some children were more receptive to learning new skills and would be determined to practice until they achieved competence:

*“She is very determined, she is very independent. Even if I try to teach her something, she would try and try until she got it right.”*

[67]

This attribute of determination was similarly reflected by some of the children, who wanted to perform activities that they perceived as challenging, such as jumping off something high or running as fast as possible:

*“Run away as fast as you can [favourite active game]”*

[69]

Conversely, other parents reported that their children would be resistant to the discomfort of new challenges that ventured beyond familiar boundaries, and would grow frustrated and withdraw ascent if they were pushed or could not immediately perform the skill:

*“When he gets frustrated, sometimes he just doesn’t want to do it anymore.”*

[67]

#### 3.7.3. Self-Efficacy to Teach FMS

Although several parents claimed that they felt confident in their ability to deliver basic FMS to their children, these parents did not specify how they were teaching these skills [67]. Moreover, several parents communicated a lack of confidence to teach FMS. This was emphasised by the parents who expressed that they did not know which skills would be necessary or appropriate to teach to children of this age group and that they were concerned about teaching skills to their child in the *“right way”* [73]. As a consequence, certain parents displayed a lack of self-efficacy to teach their children FMS and were failing to challenge them to develop new skills:

*“So, they’re kind of just doing stuff that they know how to do and stuff they can teach themselves to do.”*

[67]

A handful of parents expressed apprehension at the thought of having to teach *“sports skills”* and said that when the time came, they would ask someone else to do it or sign them up for a sports club to be professionally coached [67]. In one focus group discussion, an educator expressed an interest in developing the children’s FMS further. This was received with positivity by the parents who were pleased for the preschool staff to do so because *“it would take the pressure off me having to ensure their levels (FMS and PA) were good enough”* [86] at home. Although, parents presumed that all educators *“have a basic, yet good understanding of PA”*, with one parent believing that staff *“are all NVQ trained, and some are working up to degree level”* [86]. An educator sought to clarify this by explaining that training standards differed between settings and staff were *“not all trained”* [86]. They continued by explaining that staff were always *“willing to accept training”*, but said *“I feel there is no training available to participate in”* and *“it comes down to money”* [86].

### 3.8. The Home Environment

#### 3.8.1. Screen Time

According to children’s drawings of their favourite activities, many of them showed a preference for sedentary indoor activities at home [75]. These children tended to draw themselves playing in isolation and technology was heavily depicted within these images as their preferred activity:

*“I just like watching TV [at home], it’s my favourite watching TV.”*

[69]

*“At home I only watch TV at home and sometimes I draw.”*

[69]

*“I play Mario Kart on my iPad [at home].”*

[69]

*“TV tell me stories.”*

[75]

*“[PlayStation]…I can shoot.”*

[75]

Some of the other sedentary activities that were drawn involved various impersonation games, which were represented by children playing with dolls, toy cars, and figures of familiar cartoon characters [75]. When the children were asked why they chose these activities, these choices were similarly motivated by technology. One child explained

*“[Spiderman]…because I watch it on TV, and I want to play like them.”*

[75]

One of the children was so influenced by their screen time at home that they wanted to have further access during their time in childcare:

*“I would like to watch more movies [at kindergarten].”*

[69]

Correspondingly, educators reported that in their view children tended to stay in their houses engaging with technology instead of going outside and being active [85]. Educators recognised the increasingly sedentary lifestyle that both adults and children lead in modern society, and indicated that this may partly be explained by a cultural shift towards a reliance on technology:

*“Children [have] more access to tablets, computers,* etc. *I think culturally things have changed in terms of playing outside…kids have maybe got a tablet, and you don’t move on a tablet, do you? It’s just the way people parent has changed hasn’t it, because life changed.”*

[68]

Another educator offered further evidence of this reliance on technology by explaining how screen time was sometimes used by parents to keep children occupied while they were busy:

*“Parents have chores to complete, so sometimes the children are put in front of the television.”*

[86]

Parents did acknowledge that they often allowed their children to engage with technology, but also stated that they would make a concerted attempt to create a balance between screen time and PA by telling their children to *‘go outside and play’* [67] as an alternative to this:

*“I do let them play on their tablets and watch TV but then I’m like, ‘Alright, you guys need to go play outside too’.”*

[67]

#### 3.8.2. Restrictive Rules

Parents would occasionally set household rules to protect the property and to ensure the safety of their children, but this would inadvertently restrict access to PA opportunities:

*“We are on the second floor. We try to keep indoor activity limited. We don’t send her outside by herself.”*

[67]

Notably, some parents stated that they were happy for their children *“to be active on trampolines, kicking a football, or doing somersaults”* outdoors; however, they did not promote activities of this nature inside *“because it would involve the children breaking something or injuring themselves.”* [86]

Children had an awareness of parents’ rules and highlighted how these sometimes acted as a barrier to them participating in their favourite activities. Examples of rules that children gave included no running inside the house in case they damaged family possessions or injured their siblings:

*“No, we can’t play those games inside [referring to playing tag with friends] we knock things over.”*

[75]

*“We can’t play tag because of pushing my brother and because we are not allowed to run inside.”*

[69]

Children also communicated examples of how household rules may reach beyond the home domain and impact their play opportunities in the childcare setting. According to the children, they had been warned by their parents that activities involving *“running and sweating”* were not safe, and consequently they would choose sedentary activities instead that seemed to be more acceptable to their caregivers [75].

*“Mom says I can play with the ball with my sister, but not with all my friends and not in the kindergarten, because she is afraid I can get hurt.”*

*“No, my mum does not want me to play outside at kindergarten, because otherwise I get sick.”*

*“No, once a friend of mine pushed me and I was hurt, and my mom told my teacher that I had not to play running.”*

[75]

#### 3.8.3. Outdoor Play

In terms of instigators for FMS, it was felt by both parents and educators that spacious outdoor environments promoted *“running around”* and *“climbing and drama, which involve big movements”* [86]. This viewpoint aligned with many of the children’s perspectives, whose drawings of outdoor activities were most likely to be active ones [69]. One little boy disclosed that he liked outdoor play because *“I am happy when I can run”* [75]. These activities were often facilitated by toys and equipment such as balls, skates, bicycles, and hoops [75]:

*“[Referring to a drawing] playing frisbee.”*

*“[This is—referring to drawing]. Trampoline”*

[69]

Indeed, parents often spoke of how they attempted to provide their children with access to sports equipment at home to create a conducive environment to support FMS learning [67]. Likewise, some parents described intentionally taking out a piece of equipment to support an activity and initiate their child’s practice of a variety of skills [73]. However, although equipment featured prominently in many children’s drawings, several children drew contrasting activities that required minimal or no equipment to play:

*“[Favourite activity] Doggy, doggy, where’s your bone? [Likes it because] you get the bone, and you have to try and find it.”*

*“Pirate, police, and chasing [favourite active game].”*

[69]

When asked to explain these choices, children stated that they enjoyed this type of activity because it allowed them to use their own imaginations:

*“Because it’s like you are in a jungle…because I can see lots of animals and pretend, and we have lots of crocodiles and sometimes I pretend that there is a bridge that I have to climb over and there are crocodiles like Peter Pan.”*

[69]

*“Because we get to pretend that we are big dinosaurs and little dinosaurs, and we can chase each other.”*

[69]

Despite outdoor space being considered a facilitator of PA and FMS by all stakeholders, educators shared their concerns that some home environments lacked the scope for children to be active, with some families living in small houses with little outdoor space or in *“flats without gardens”* [85]. These apprehensions were echoed by a selection of children who were unable to play their favourite games at home because they were restricted by space:

*“It’s too small [at home to play tag].”*

[69]

#### 3.8.4. Social Interactions

Social participation was a substantial feature of children’s favoured activity choices and seemed to significantly influence their choice to engage in certain activities. This was evidenced in their drawings, with several children focusing on who they were playing with rather than the activity itself. Siblings were frequently involved in the chosen activities:

*“[Plays with] my sister…Lennox, my brother.”*

[69]

*“[Ball]…I play with my brother…I play with my sister.”*

[75]

Equally, the availability of peers proved to be an important aspect of children’s play:

*“Well, I like playing tag with my friends.”*

[69]

*“[Football]…I have fun playing with my friends.”*

[75]

*“I have a lot of fun with my friends, and I feel better.”*

[75]

It was felt by parents that *“as a kid, playing together with balls and running around and stuff is a really big social thing”* [67]. Therefore, they embraced socialisation by actively taking their children to a place of play, such as a park, open spaces, or a family member’s house, and gave their children further PA opportunities by signing them up for sports [67]. One boy revealed that the reason he enjoyed playing football was because he had been given the opportunity by his parents to *“go to football lessons”* [75]. However, parents were often disclosed by children as the core reason for why they found enjoyment in a particular activity:

*“[Likes the activity because] Mum usually pushes us up on the trampoline.”*

*“[Likes the activity because] Because I get to play with Mumma and dada.”*

*“[Likes the activity because] Because my dad plays.”*

[69]

#### 3.8.5. The Impact of Deprivation

There was widespread recognition from educators of the impact of socioeconomic status on children’s FMS and PA opportunities. One of the educators stated that *“less children at my school where I am now take part in extracurricular sporting activities”* [68]. Financial constraints and deprivation were frequently mentioned by educators as a barrier to socialisation, clubs, and extracurricular activities. An example of this was shared by one educator who reported how parents would show an interest in their children attending school clubs, but when they became aware that the club had a fee attached to it they would ultimately decline attendance [85].

*“80% of our children live in the poorest 20% of postcodes in the UK…parents can’t afford to take them to do extra-curricular things.”*

[68]

*“[Parents] can’t pay for it, they simply don’t have the money.”*

[85]

Parents added that *“when the weather is not good, it is costly for swimming, play pits, and structured activities”*, but felt that if their families had more disposable income, or if certain activities were cheaper or free, then they would be able to promote PA and FMS development more with their children [86]. In addition to finances, educators told of their experiences with families who restricted their children’s activity in the community out of fear regarding neighbourhood safety:

*“Some parents, because of the area and there’s different cultures, there’s a wide range of cultures in this area, maybe the fear of even letting them go outside.”*

[85]

*“They [children] will say that oh ‘parents don’t take me’, ‘there are dogs there’.”*

[85]

## 4. Discussion

To the best of our knowledge, this was the first study to systematically synthesise the qualitative literature reporting the determinants of family engagement in 2–6-year-old children’s FMS practice. The key findings show that parents often undervalue FMS and lack the self-efficacy to teach these skills due to poor underlying knowledge and understanding. Secondly, parents are a major source of children’s enjoyment of activities and can thus positively influence their practice of FMS through role-modelling and co-participation. Thirdly, the home environment can be a facilitator for children’s FMS, but this is impacted by deprivation and modern reliance on screen time. Importantly, the data analysed in this review were drawn from a pool of research that was of a high quality overall, providing greater weight to the findings.

Parents frequently undervalued FMS and this diminished their support for skill practicing with their children. In some instances, this was due to conflicting family priorities. An example of this was parents’ prioritisation of play over structured teaching. Children also indicated that their preference was for autonomy and unstructured activities that had no learning objectives. Research has offered justifications for free play [87], but far superior gains in FMS have been elicited under instructed conditions [88,89,90,91]. However, it has been argued that FMS practice should be underpinned by the pedagogy of play, which may improve children’s motivation to learn new skills compared with being taught in an isolated and decontextualised manner, which is often less enjoyable [92]. This may be particularly important for the children in this study who were reported by their parents as having difficult temperaments and resisting formal skill practice. Consequently, despite structured teaching being the most effective method of delivery for FMS, a balance between instruction and play is needed to facilitate enjoyment and engage families in FMS.

A further conflicting priority was parents’ prioritisation of academic rigour over FMS. Cases of explicit focus on educational attainment at the expense of PA and motor development are similarly present within the literature [43,93,94]. Yet, these views are at odds with research that has postulated that a positive synergy may exist between FMS, cognition, and academic learning [95]. Despite a small number of studies describing inconsistent or weak evidence in favour of this interaction [96,97], the overwhelming consensus is that greater FMS ability in children is significantly associated with higher academic performance [98,99,100,101,102]. Therefore, parents may better serve their children academically, as well as physically and mentally, by reprioritising support for their motor development. However, if parents lack the knowledge and awareness of the importance of providing FMS support, their engagement is unlikely to improve in practice.

Overall, parent knowledge and understanding of FMS was limited, and this appeared to be an underlying cause of the lack of parental value and support of motor development in this study. This was illustrated by parents who assumed that these skills were acquired naturally, and parents who did not realise that these skills could be acquired at such a young age. These findings are comparable to similar research that cited a lack of parental awareness as having a detrimental impact on engagement in practice at home [103]. In the UK, recommendations that include advice on appropriate motor activities for EY children are readily available and accessible to parents via the UK Chief Medical Officers’ PA guidelines [104]. However, the UK guidelines have historically failed to include a communication and implementation plan to effectively disseminate information into the public domain [105]. Further, healthcare professionals who were entrusted to disseminate guidance to the UK public have admitted to having a lack of time, knowledge, and confidence to discuss PA with patients [106,107], as well as a lack of centrally available resources for promoting PA [108]. These factors could contribute to parents feeling insufficiently informed and supported. Given that EY children spend much of their time at home where they are primarily influenced by their parents [43], this apparent knowledge gap may partly explain the ongoing secular decline in children’s FMS, despite the plethora of interventions aimed at EYs education [19].

There was disagreement amongst participants over whose obligation it was to promote FMS to parents. Notably, certain parents who lacked the self-efficacy to teach FMS were happy to pass teaching responsibilities over to educators on the assumption that they were fully qualified and capable to do so. Certainly, schools and preschools are considered ideal settings for the promotion of FMS as they can provide quality facilities, equipment, and professional teaching personnel [85,109]. However, formal screening and assessment of FMS are not common practice in schools in the UK [110], and according to recent surveys, only 15% of educators felt they had the confidence and capability to teach FMS [110] and almost one in two had zero comprehension of the term FMS [111]. This is concerning given that parents may be disengaging at home under the misconception that children are adequately supported in schools. This issue is further complicated by chronic underfunding in schools [27], insufficient teacher training in PA [112], and competing pressures to deliver the core curriculum with heavy workloads for teachers [110]. In consideration of this, it does not seem appropriate that promoting FMS to families should fall primarily to educators. This would further increase their workload and they themselves may not be best placed to do so due to their own struggles with capability. However, it is clear there is a need for more congruent teacher–parent collaboration, and for more effective knowledge exchange between all relevant stakeholders beginning at policy level. This would ensure adequate knowledge and guidance is filtered down to families to improve the application of FMS in homes.

Educators suggested that children with active parents were more likely to be physically able. Parental activity participation that is observable to children may be referred to as parent role-modelling [41] and is accepted as a positive influence on children’s PA and FMS [63,113,114]. This may be reinforced by co-activity [115], which was an approach utilised by parents in the current study which also resonated with children. Notably, FMS interventions that have incorporated parent–child co-activity have produced significant outcomes for FMS [44]. Alternatively, it has been proposed that positive parenting practices, such as praise, encouragement, and reinforcement, may be a superior form of support to children as this increases their feelings of competence and behavioural intentions towards PA [41]. However, such practices are reliant on parental self-efficacy to execute tasks with their child [116], as well as relying on children to have suitably agreeable and emotionally regulated temperaments that are conducive to learning [117]. This is interesting as confident parents in this review frequently used demonstrations and reasoned communication to promote FMS to their children and recognised how individual child temperaments and needs may influence learning. Accordingly, it may be surmised that parents who directly engage with their children’s PA can positively influence their FMS via both role modeling and support, but the choice of approach should be flexible to suit individual attributes and family needs.

The outdoor space, availability of equipment, and socialisation were all considered to be facilitators of family FMS engagement. This corresponds with research that has emphasised the need for enriching home environments, resources, and family and social connections, all of which support healthy growth of motor skills [54,60,63,118]. However, access to these environments was often restricted by factors relating to deprivation and neighbourhood safety in the current study. Indeed, it is well known that children living in lower-income communities are at greater risk of physical inactivity and poor FMS development [27,119,120]. Echoing the views of stakeholders, this negative relationship has been attributed to the lack of safe open spaces [121,122], and the poor availability of activity resources and access to extracurricular activities due to low family income [121]. It is clear from the evidence that deprivation may negatively impact family engagement in PA. Therefore, more community initiatives are needed to provide safe spaces for PA in neighbourhoods and affordable access to clubs and extracurricular activities for children living in deprived areas. Although, interestingly, the preferred activities of many of the children in this review involved using their imagination and required little to no equipment. This suggests that parents with a low income may also benefit from further education on ways to engage in activities with their children at minimal cost.

An additional barrier to PA and FMS within the home environment was the influence of technology. Consistent with similar qualitative studies [82,113,122], child participants indicated a strong preference for sedentary screen-based activities which educators felt demonstrated a cultural shift towards technology in modern parenting styles. This mirrors research that has documented parents regularly co-viewing television with children [41], using it to distract children while they are busy [122], and to comfort children when they are upset [82]. Screen time is a prevalent way in which children accumulate sedentary hours [64] and is thus inversely associated with children’s FMS [64,123,124,125]. This is also concerning as parenting behaviours appear to be promoting screen time to their children rather than discouraging it. Furthermore, in the UK there are currently no official governmental recommendations on children’s daily screen viewing [82], so this guidance is urgently needed for UK families. However, parents can distinguish between educational screen time versus entertainment and can be supportive of intervention delivery via mobile devices [126]. This concept has seen the recent emergence of several family-focused digital interventions that have significantly improved the FMS of children [48,49,127]. Thus, despite the negative connotations surrounding screen time, the ubiquitous use of mobile devices in family routines may present a promising opportunity to promote FMS to families through a suite of interventions.

### 4.1. Limitations and Strengths

As with all studies, there were limitations to this review. The viewpoints provided by children were from active play due to them being developmentally unable to articulate on FMS, so they could be considered only indirectly relevant to FMS. Moreover, young children’s illustrations and explanations can be challenging to interpret. Consequently, the conclusions drawn from children in this study should be treated with caution. A further limitation was the small number of studies included in this review due to the paucity of qualitative research related to FMS in the family context, which restricted the amount of data for analysis. The limitations were equally balanced by the strength of the research. This review reflected on a holistic collection of key stakeholder perspectives, which were extracted from high-quality research and offered rounded discussion on the determinants of family FMS practice. The inclusion of children, who are central to the phenomenon, added valuable additional insight and context to the adult perspectives provided. A final strength was the specific consideration of FMS. PA behaviours have been extensively studied previously, but FMS are often overlooked as an outcome. Therefore, the findings of this review make a valuable contribution to the literature and in practice.

### 4.2. Practical Implications

Given the paucity of research focused on FMS in the family context, and the lack of understanding of the determinants that may impact family engagement in FMS practice, it may be beneficial to disseminate the key findings from this research through appropriate professional networks. For example, connections could be made with Active Derbyshire and Active Notts, who through the Making Our Move Campaign [128] aim to share opportunities and empower communities in the East Midlands region to be more physically active. Active Derbyshire and Active Notts are supported by Sport England and form one of 43 Active partnerships across the country. Collaboration with these networks may help facilitate dialogue with stakeholders both locally and nationally to inform the development of future interventions, best practice, and policies to promote FMS to families. Further, the Active Partners Trust is committed to tackling inequality and increasing diversity in PA engagement so these networks will be used to reach diverse cultural groups, allowing findings to be applied in different cultural contexts. Moreover, the current study has highlighted a concerning gap in the research regarding families that could be addressed by future investigations. Therefore, it may be important for the findings of this review to be presented to organisations such as the International Motor Competence Network (IMCNetwork) [129] and the International Motor Development Research Consortium (I-MDRC) [130], which involve collaborations between researchers and academics to enhance the promotion and translation of global knowledge regarding motor development research.

## 5. Conclusions

Parents are the main source of enjoyment in children’s PA participation and can effectively promote FMS to children through role-modelling and implementing positive parenting support practices. However, parental knowledge of FMS is currently limited, and many parents lack the self-efficacy to teach skills to their children. Therefore, there is a need for stronger teacher–parent partnerships and greater knowledge exchange between all relevant stakeholders, beginning at policy level, to ensure guidance and support is effectively filtered down to families. The family home can provide an enriching environment that supports the growth of children’s motor competence, but modern family dependence on screen time, as well as factors related to deprivation and neighbourhood safety, may inhibit children’s PA participation. Further community initiatives are required to facilitate more inclusive access to open spaces, facilities, and resources to increase family engagement with PA and FMS practice.

## Figures and Tables

**Table 1:** Qualitative study characteristics and quality assessment.

Author and Country	Population and Characteristics	Data Collection	Analysis Method	Outcomes and Recommendations	MMAT Quality
Agard et al. (2021) [67], USA.	31 parents: 26 mothers, 5 fathers. 20 Caucasian, 11 Hispanic. Heterogeneous SES.	Semi-structured in-person interviews.	Directed content analysis.	Parents lacked knowledge and awareness of FMS, leading to them overlooking the importance of supporting children’s FMS. Parent involvement in children’s PA, positive parenting practices, and social support may enrich parent–child interactions and enhance children’s FMS. Child temperament should be considered to help tailor future interventions.	*****
Eyre et al. (2022) [85], UK.	8 reception teachers, 100% female. Area of high deprivation and ethnic diversity.	Focus group discussions in-person.	Inductive thematic analysis.	Collaborative working across disciplines and sectors is needed to address the multitude of barriers that negatively impact children’s PA opportunities and motor development in areas of deprivation and ethnic diversity. Researchers, policymakers, and stakeholders must work as collective workforces to target all levels of influence on children’s PA and FMS.	*****
Roscoe et al. (2017) [86], UK.	17 preschool staff and parents: 10 parents, 7 staff. 100% female. 16 Caucasian, 1 South Asian. Area of high deprivation.	Focus group discussions in-person.	Thematic analysis.	The outdoor environment is a major influence on children’s PA opportunities in areas of high deprivation. More resources are required to ensure adequate PA participation in children. The home environment needs to become more supportive in terms of PA and FMS promotion, and this may be facilitated through parent involvement and training to better engage them in low-cost activities.	*****
Dobell et al. (2021) [68], UK.	12 Educators: 2 headteachers, 7 teachers, 3 external PE providers. 9 females, 3 males. Homogeneous SES.	Semi-structured online interviews.	Thematic analysis.	Educators face significant multifaceted barriers when supporting children’s PA and FMS, including inadequate parental involvement at home. To improve FMS tuition in children, further intervention, staff training, parental education, and resources are required, but consideration of cost is important in areas of high deprivation.	*****
James et al. (2024) [73], Canada.	20 parents: 10 female, 10 male. 3 parent dyads (1 dyad completed together). Mean age: 41 years. Preschoolers discussed: 50% female, 50% male.	Semi-structured online interviews.	Deductive and inductive thematic analysis.	Parents are less intentionally supportive of FMS compared to PA. Interventions to improve parents’ own PA identity and physical literacy, and their understanding of the importance of FMS, may help progress parent support behaviours and children’s motor development. The provision of behaviour regulation strategies related to the planning and monitoring of PA and FMS may translate the intention to support FMS into behaviour.	*****
Wiseman et al. (2019) [69], Australia.	29 preschool children: 15 girls, 14 boys. Mean age: 4.28 years.	Draw and tell	Inductive thematic analysis.	Considering children’s own views on active play preferences and associated determinants can enhance the promotion and enjoyment of active play in early childhood. Children desire control over activity choices and prefer unstructured and imaginative free play that they perceive as challenging. Parents may be overestimating their children’s activity levels away from the home, leading to acceptance of sedentary choices at home.	*****
Cammisa et al. (2011) [75], Italy.	49 preschool children: 23 girls, 26 boys.	Draw and tell	No disclosed method. Subjective summarisation.	Parental perception of the risk of injury or illness can restrict activity opportunities for children. Future strategies should balance children’s preferences with risk aversion. Children who are exposed to a sedentary lifestyle become accustomed to it and are less likely to seek active play or be stimulated by it. There is a need to change the beliefs and behaviours of both parents and teachers who have created “invisible” barriers for children.	***

FMS, fundamental movement skills; MMAT, mixed-methods appraisal tool; PA, physical activity; PE, physical education; SE, socioeconomic status; UK, United Kingdom; USA, United States of America. Study quality was assessed using MMAT and is reported using asterisks (*) to describe the quality rating of the article, ranging from 1*, where 20% of the quality criteria have been met, to 5*****, where 100% of the quality criteria have been met [79].

**Table 2:** Summary of themes, subthemes, and quotes associated with the determinants of family FMS practice from a multistakeholder perspective.

Themes	Subthemes	Interview Extract Examples
Parent beliefs and understanding of FMS	Value assigned to FMS	*“I want him to be able to have those opportunities [speaking about physical activities] and not just sit down and watch people and be like ‘well, I can’t do that’ or ‘I didn’t know how to do that’.”* [73] *“Not really [important] unless my child was obese, and I needed to do something about it.”* [86]
	Conflicting priorities	*“She turns round to me and says, ‘my mum says I don’t need friends and I don’t need sport to get into Oxford’.”* [68] *“[parents] they’re busy, the dad is always at work, never there and the mum they tend to have quite a lot of children so they’re at home on their own with these children.”* [85]
	Knowledge and promotion of FMS	*“…my background knowledge is not that good on this area…I feel nursery staff need to help me promote this.”* [86] *“It’s lots of parents who really want to do the best thing for their child, but they also won’t do anything that upsets their child. So, it’s… about educating and promoting, but not making parents feel bad about it”* [68]
Parenting practices	Positive approaches and engagement with FMS	*“And we were like, showing her how to do it. ‘Can you stand like this; you go like this.”* [73] *“Any sort of success I think should be celebrated, especially when a child is uncomfortable with their competence and their skills.”* [67]
	Awareness of child temperament	*“She is very determined; she is very independent. Even if I try to teach her something, she would try and try until she got it right.”* [67] *“Every child is individual.”* [68]
	Self-efficacy to teach FMS	“*So they’re kind of just doing stuff that they know how to do and stuff they can teach themselves to do*.” [67] *“It would take the pressure off me having to ensure their levels (FMS and PA) were good enough.”* [86]
The home environment	Screen time	*“I just like watching TV [at home], it’s my favourite watching TV.”* [69] *“Children [have] more access to tablets, computers,* etc. *I think culturally things have changed in terms of playing outside.”* [68]
	Restrictive rules	*“We are on the second floor. We try to keep indoor activity limited. We don’t send her outside by herself.”* [67] *“Mom says I can play with the ball with my sister, but not with all my friends and not in the kindergarten, because she is afraid I can get hurt.”* [75]
	Outdoor play	*“Because it’s like you are in a jungle…because I can see lots of animals and pretend, and we have lots of crocodiles and sometimes I pretend that there is a bridge that I have to climb over and there are crocodiles like Peter Pan.”* [69] *“I am happy when I can run.”* [75]
	Children’s social networks	*“…as a kid, playing together with balls and running around and stuff is a really big social thing.”* [67] *“I have a lot of fun with my friends, and I feel better.”* [75]
	The impact of deprivation	*“80% of our children live in the poorest 20% of postcodes in the UK… and a lot of the parents have got lots of fears about going outside”* [68] *“Some parents, because of the area and there’s different cultures, there’s a wide range of cultures in this area, maybe the fear of even letting them go outside.”* [85]

## Data Availability

No new data were created or analysed in this study. Data sharing is not applicable to this article.

## Appendix A

**Table A1:** MMAT quality assessment outcomes [79].

First Author	Qualitative Studies					Randomised Controlled Trials					Non-Randomised Studies					Quantitative Descriptive Studies					Mixed-Methods Studies					MMAT Quality	Comments
	1.1	1.2	1.3	1.4	1.5	2.1	2.2	2.3	2.4	2.5	3.1	3.2	3.3	3.4	3.5	4.1	4.2	4.3	4.4	4.5	5.1	5.2	5.3	5.4	5.5		
Agard [67]	1	1	1	1	1																					*****	No issues identified
Eyre [85]	1	1	1	1	1																					*****	No issues identified
Roscoe [86]	1	1	1	1	1																					*****	No issues identified
Dobell [68]	1	1	1	1	1																					*****	No issues identified
James [73]	1	1	1	1	1											1	1	1	1	1	1	1	1	1	1	*****	No issues identified
Wiseman [69]	1	1	1	1	1																					*****	No issues identified
Cammisa [75]	1	1	0	0	1																					***	1.3, 1.4. No discernible method of analysis or interpretation.

Study quality was assessed using the mixed-methods assessment tool (MMAT) and is reported using asterisks (*) to describe the quality rating of the article, ranging from 1*, where 20% of the quality criteria have been met, to 5*****, where 100% of the quality criteria have been met [79].
